# Characteristics of methicillin-resistant *Staphylococcus aureus* isolates from bovine mastitis milk in South Korea: molecular characteristics, biofilm, virulence, and antimicrobial resistance

**DOI:** 10.1128/spectrum.01197-24

**Published:** 2024-10-22

**Authors:** Hye Jeong Kang, Ju-Yeon You, Seung Hoe Kim, Jin-San Moon, Ha-Young Kim, Jae-Myeong Kim, Young Ju Lee, Hyun-Mi Kang

**Affiliations:** 1 Animal and Plant Quarantine Agency, Gimcheon, South Korea; 2 College of Veterinary Medicine and Institute for Veterinary Biomedical Science, Kyungpook National University, Daegu, South Korea; Ross University School of Veterinary Medicine, Basseterre, Saint Kitt and Nevis

**Keywords:** MRSA, bovine mastitis, PVL, TSST-1, ST22, biofilm, virulence, genotyping, antimicrobial resistance

## Abstract

Methicillin-resistant *Staphylococcus aureus* (MRSA) is a drug-resistant pathogen causing subclinical and clinical bovine mastitis. This study examined the molecular properties, biofilm formation, virulence genes, and antimicrobial susceptibility of MRSA isolates from mastitis-infected dairy cow milk in South Korea. Whole-genome sequencing of an ST22-SCC*mec* IV MRSA strain positive for Panton–Valentine leukocidin (PVL) and toxic shock syndrome toxin-1 (TSST-1) was also performed. Of the 488 *S*. *aureus* isolates, 30 (6.1%) were identified as MRSA, harboring the *mecA* gene. MRSA exhibited the highest resistance to kanamycin (66.7%) among non-beta-lactam antibiotics. Multidrug resistance was observed in 83.3% of MRSA isolates. All MRSA strains had the capacity to form biofilm and harbored biofilm-related genes. The primary virulence genes included *hla*, *hlb*, *lukED*, *seg*, *sei*, *sem*, *sen*, and *seo*. The ST72-t324-SCC*mec* IV genotype was the most prevalent. Of note, three ST22-SCC*mec* IV isolates were positive for PVL and TSST-1. Our findings suggest that the majority of MRSA isolates from milk obtained from dairy cows with mastitis are multidrug-resistant, capable of forming robust biofilms, and harbor multiple virulence genes. The presence of PVL- and TSST-1-positive ST22-SCC*mec* IV isolates in mastitis-infected bovine milk highlights the need for persistent monitoring to mitigate possible public health risks.

**IMPORTANCE:**

This study reports on the presence and characteristics of methicillin-resistant *Staphylococcus aureus* strains in milk from mastitis-infected cows. To our knowledge, this is the first report of a Panton–Valentine leukocidin- and toxic shock syndrome toxin-1-positive ST22-SCC*mec* IV strain in South Korea.

## INTRODUCTION

*Staphylococcus aureus* frequently causes infectious bovine mastitis in many countries (1–3). *S. aureus* has a substantial impact on mastitis development and represents a public health concern owing to its multiple virulence factors, including biofilm, enterotoxins, toxic shock syndrome toxin (TSST), hemolysins, and leukotoxins (1, 4). *S. aureus* is also known for its capacity to acquire antibiotic resistance (5), with methicillin-resistant *S. aureus* (MRSA) being a prevalent bacterium worldwide (3). The emergence of MRSA in the milk of dairy cows with mastitis represents a public health concern owing to the difficulty involved in its treatment along with the potential risk of transmission to humans (6). MRSA strains have a modified penicillin-binding protein, with a low affinity to most beta-lactam antibiotics. Notably, methicillin resistance is imparted by *mecA*/*mecC* genes, located in the staphylococcal cassette chromosome (SCC) element (3, 7). Three distinct types of MRSA strains—healthcare-associated MRSA (HA-MRSA) in the 1960s, community-acquired MRSA (CA-MRSA) in the 1990s, and livestock-associated MRSA (LA-MRSA) in the 2000s—have emerged (8).

In South Korea, bovine mastitis is primarily caused by *S. aureus*, and several studies have reported the presence of MRSA isolates in milk from cows with mastitis (6, 9–11). These MRSA isolates contain a variety of enterotoxins and Panton–Valentine leukocidin (PVL), a two-component toxin that causes pore formation in the leukocyte cell membrane, with a potency 100-fold greater than that of other leukotoxins (4, 6, 11). ST72 SCC*mec* IV, the major community-associated MRSA clone in South Korea, is the most common MRSA isolate in dairy cows with mastitis (10–12). MRSA has been repeatedly detected in milk from cows with mastitis in South Korea, along with new genotypes associated with community- or hospital-acquired human MRSA (6, 11).

Understanding the molecular epidemiology of MRSA is critical for developing effective preventive measures (8). Molecular typing of *S. aureus* is epidemiologically valuable for tracing outbreaks, assessing the source of colonization (e.g., livestock- or human-associated), and distinguishing between community and hospital strains (5). Traditional approaches for identifying MRSA lineages and strains include pulse-field gel electrophoresis (PFGE), multilocus sequence typing (MLST), SCC*mec* typing, and staphylococcal protein A (*spa*) typing (5, 8). Whole-genome sequencing has recently been demonstrated to be a reliable method for accurately identifying DNA variations in organisms, making it a useful tool for tracking MRSA transmission pathways in hospitals and communities (5).

This study aimed to investigate the molecular characteristics, biofilm formation, virulence genes, and antibiotic resistance of MRSA isolates from mastitis-infected dairy cow milk in South Korea. Further, a comparative genomic analysis of a PVL- and TSST-1-positive ST22-SCC*mec* IV MRSA strain through whole-genome sequencing was conducted.

## RESULTS

### Antimicrobial resistance in MRSA

Phenotypic and genotypic antimicrobial resistance tests on MRSA isolated from milk samples of cows with mastitis were carried out to characterize their antimicrobial resistance profiles. Notably, of the 488 *S*. *aureus* isolates, 30 (6.1%) were resistant to oxacillin + 2% NaCl and were classified as MRSA (Table 1). The distribution of MRSA was highest in 2019, at 10.6%, followed by 8.5% in 2017, 8.1% in 2018, 5.1% in 2022, 5.0% in 2021, and 1.2% in 2020. The phenotypic antimicrobial resistance profiles of methicillin-sensitive *S. aureus* (MSSA) and MRSA strains are presented in Table 2. MRSA isolates are extremely resistant to ampicillin, ceftiofur, oxacillin + 2% NaCl, and penicillin, with resistance ranging from 90.0% to 100%, whereas MSSA isolates are resistant to these antimicrobial agents to a lesser extent (0–36.7%) (*P* < 0.05). MSSA (30.3%) isolates showed higher resistance rates against sulfadimethoxine than MRSA isolates (3.3%) (*P* < 0.05). Both MSSA and MRSA isolates exhibited little or no resistance (0–3.3%) to cephalothin, penicillin/novobiocin, pirlimycin, and tetracycline. Among the non-beta-lactam antibiotics, MRSA isolates were most frequently resistant to kanamycin (*n* = 20, 66.7%), followed by ciprofloxacin (*n* = 4, 13.3%), gentamicin (*n* = 4, 13.3%), and trimethoprim (*n* = 3, 10.0%) (*P* < 0.05). Most MRSA strains, however, were sensitive to sulfamethoxazole, vancomycin, clindamycin, linezolid, chloramphenicol, mupirocin, fusidate, and quinupristin/dalfopristin. In addition, 25 MRSA isolates (83.3%) exhibited multidrug resistance, with resistance to at least three antibiotic classes.

**TABLE 1:** Distribution of MRSA isolated from quarter milk samples of cows with mastitis in South Korea (2017–2022)

Year	No. of isolates		Distribution of MRSA (%)
	*Staphylococcus aureus*	MRSA	
2017	59	5	8.5
2018	62	5	8.1
2019	85	9	10.6
2020	84	1	1.2
2021	119	6	5.0
2022	79	4	5.1
Total	488	30	6.1

**TABLE 2:** Phenotypic resistance profiles of *Staphylococcus aureus* isolated from the milk of cows with bovine mastitis in South Korea^*a*^

Antimicrobial class	Antimicrobial agent	Breakpoints	No. of resistance isolates (%)	
			MSSA (*n* = 458)	MRSA (*n* = 30)
Aminoglycoside	Kanamycin	≥64	NT	20 (66.7)^b^
	Gentamicin	≥16	NT	4 (13.3)^c^
Cephems	Cefoxitin	≥8	NT	30 (100)^a^
	Ceftiofur*	≥8	89 (19.4)^a^	27 (90.0)^ab^
	Cephalothin	≥32	1 (0.2)^b^	0 (0)^c^
Sulfonamides	Trimethoprim	≥16	NT	3 (10.0)^c^
	Sulfadimethoxine*	≥512	139 (30.3)^a^	1 (3.3)^c^
	Sulfamethoxazole	≥512	NT	0 (0)^c^
Glycopeptide	Vancomycin	≥16	NT	0 (0)^c^
Lincosamides	Clindamycin	≥4	NT	0 (0)^c^
	Pirlimycin	≥4	3 (0.7)^b^	0 (0)^c^
Macrolides	Erythromycin	≥8	99 (21.6)^a^	3 (10.0)^c^
Oxazolidinones	Linezolid	≥8	NT	0 (0)^c^
Penicillins	Ampicillin*	≥0.5	162 (35.4)^a^	30 (100)^a^
	Oxacillin + 2% NaCl*	≥4	0 (0)^b^	30 (100)^a^
	Penicillin*	≥0.25	168 (36.7)^a^	30 (100)^a^
Penicillins/aminocoumarin	Penicillin/novobiocin	≥4/8	1 (0.2)^b^	0 (0)^c^
Phenicols	Chloramphenicol	≥32	NT	1 (3.3)^c^
Psedomonic acid	Mupirocin	≥256	NT	1 (3.3)^c^
Quinolones	Ciprofloxacin	≥4	NT	4 (13.3)^c^
Steroid	Fusidate	≥2	NT	0 (0)^c^
Streptogramins	Quinupristin/dalfopristin	≥4	NT	0 (0)^c^
Tetracyclines	Tetracycline	≥16	15 (3.3)^b^	1 (3.3)^c^

^*a*^ NT, Not tested. Asterisks indicate that the resistance rates of MSSA and MRSA isolates are significantly different (P < 0.05). Values with different lowercase superscript letters (a–c) represent significant differences in resistance rates to each antimicrobial agent (*P* < 0.05).

**Fig 1:**
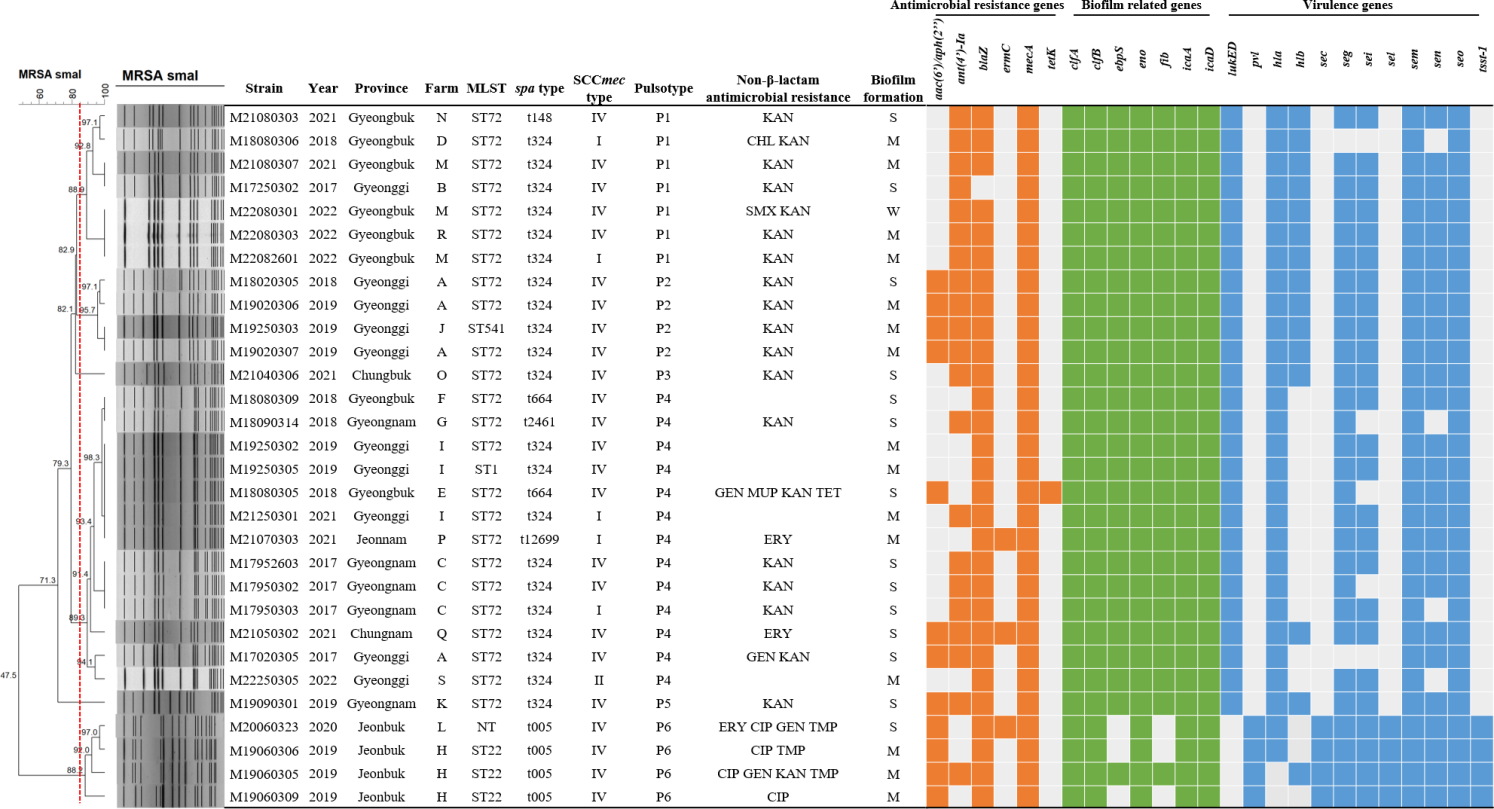
Dendrogram of the PFGE-*SmaI* patterns, molecular characterization, antimicrobial resistance, antimicrobial resistance genes, biofilm-related genes, and virulence genes of 30 MRSA isolated from cows with bovine mastitis in South Korea. NT, untypable; KAN, kanamycin; CHL, chloramphenicol; SMX, sulphamethoxazole; GEN, gentamicin; MUP, mupirocin; TET, tetracycline; ERY, erythromycin; CIP, ciprofloxacin; TMP, trimethoprim; S, strong; M, moderate; and W, weak.

The antimicrobial resistance gene profiles of MRSA isolates are shown in Fig. 1. All MRSA isolates harbored the methicillin resistance gene *mecA*, and all except one harbored the penicillin resistance gene *blaZ*. Aminoglycoside resistance genes *ant(4′)-Ia* and *aac(6′)-Ie + aph(2″*) were identified in 70.0% (*n* = 21) and 40.0% (*n* = 12) of MRSA isolates, respectively. The macrolide resistance gene *ermC* was detected in 10.0% (*n* = 3) of MRSA isolates, whereas the tetracycline resistance gene *tetK* was detected in 3.3% (*n* = 1). All MRSA isolates were negative for *aph(3′)-IIIa*, *cfr*, *ermA*, *ermB*, *fexA*, *msrA*, and *tetM*. The most prevalent resistance gene profile among MRSA isolates was *ant(4′)-*Ia + *blaZ + mecA,* accounting for 40.0% (*n* = 12), followed by *aac(6′)-Ie + aph(2″) + ant(4′)-*Ia + *blaZ + mecA,* accounting for 23.3% (*n* = 7).

### Virulence genes in MRSA

To evaluate the pathogenicity of MRSA isolates, PCR was used to detect virulence genes. The virulence gene profiles of 30 MRSA isolates are shown in Fig. 1. The staphylococcal enterotoxin genes *sem* and *seo* were detected in all MRSA isolates. Furthermore, *seg*, *sen*, *sei*, *sec*, and *sel* were identified in 93.3% (*n* = 28), 86.7% (*n* = 26), 83.3% (*n* = 25), 13.3% (*n* = 4), and 13.3% (*n* = 4) of isolates, respectively. In contrast, no isolate harbored *sea*, *seb*, *sed*, *see*, *seh*, *sej*, *sek*, *sep*, *seq*, and *ser. tsst-1* (toxic shock syndrome toxin gene), *hla* and *hlb* (hemolysin genes), *lukED* and *pvl* (leukocidin genes) were found in 13.3% (*n* = 4), 93.3% (*n* = 28) and in 50.0% (*n* = 15), 86.7% (*n* = 26), and 13.3% (*n* = 4) of MRSA isolates, respectively. However, none of the MRSA isolates contained the exfoliative toxin A or B genes (*eta* and *etb*). All MRSA isolates harbored 5–10 virulence genes. The most prevalent virulence gene profile among MRSA isolates was *lukED + seg + sei + sem + sen + seo + hla + hlb,* accounting for 43.3% (*n* = 13), followed by *lukED + seg + sei + sem + sen + seo + hla,* accounting for 20.0% (*n* = 6).

### Biofilm formation by MRSA

The biofilm formation test indicated that all MRSA strains could form biofilms (Fig. 1). Notably, 15 (50.0%), 14 (46.7%), and 1 (3.3%) isolates demonstrated moderate, strong, and weak biofilm-forming ability.

All MRSA isolates harbored the clumping factor (*clfA* and *clfB*), the laminin-binding protein (*eno*), and the intercellular adhesion (*icaA* and *icaD*) genes. The elastin-binding protein (*ebpS*) and the fibrinogen-binding protein (*fib*) genes were detected in 90.0% (*n* = 27) of isolates. In contrast, no isolates harbored the biofilm-associated protein (*bap*), bone sialoprotein binding protein (*bbp*), collagen-binding protein (*cna*), or fibronectin-binding protein (*fnbA* and *fnbB*) genes. The most common biofilm-related genotype was *clfA + clfB + ebpS + eno + fib + icaA +* icaD, accounting for 90.0% (*n* = 27), followed by *clfA + clfB + eno + icaA +* icaD, accounting for 10.0% (*n* = 3).

### Molecular characterization of MRSA

To determine the genetic diversity of MRSA isolates, MLST, *spa* typing, SCC*mec* typing, and PFGE (Fig. 1) were used. Of the 30 MRSA isolates, 29 were typable, with four MLST types observed, whereas one isolate was not typable. ST72 had the highest prevalence (80.0%, *n* = 24), followed by ST22 (10.0%, *n* = 3), ST1 (3.3%, *n* = 1), and ST541 (3.3%, *n* = 1). Six types were observed using *spa* typing, with t324 accounting for 70.0% (*n* = 21). The remaining *spa* types were t005 (*n* = 4, 13.3%), t664 (*n* = 2, 6.7%), t12699 (*n* = 1, 3.3%), t148 (*n* = 1, 3.3%), and t2461 (*n* = 1, 3.3%). The most common SCC*mec* type was IV (*n* = 24, 80.0%), whereas types I and II were less common, accounting for 16.7% (*n* = 5) and 3.3% (*n* = 1), respectively. The ST72-t324-SCC*mec* IV genotype was the most prevalent among the 30 MRSA isolates, accounting for 46.7% (*n* = 14) of all isolates, followed by ST72-t324-SCC*mec* I (13.3%, *n* = 4), ST22-t005-SCC*mec* IV (10.0%, *n* = 3), and ST72-t664-SCC*mec* IV (6.7%, *n* = 2).

Using PFGE analysis, we identified six pulsotypes that had 85% commonality. P4 was the main pulsotype with 13 (43.3%) isolates, followed by P1 with 7 (23.3%), P2 and P6 with 4 (13.3%), and P3 and P5 with 1 (3.3%) each. P1–P5 primarily contained ST72 isolates, whereas P6 contained ST22 and untypable isolates. P1 also included isolates from Gyeongbuk, P2 included isolates from Gyeonggi, P6 included isolates from Jeonbuk, and P4 included isolates from multiple regions. Some isolates collected in different years from the same farm (Farms A, M, and I) had the same pulsotype, confirming the expansion and persistence of the same clone. Depending on the pulsotype, isolates exhibited differences in some gene patterns. For biofilm-related genes, P1, P2, P3, and P5 were positive for all nine genes tested, and P4 was positive for all genes except *hlb*. For virulence genes, only the P6 isolates were positive for *pvl*, *sec*, *sel*, and *tsst-1*. In contrast, only P1–P5 isolates were positive for *lukED*.

### Comparative phylogenetic analysis of ST22-SCC*mec* IV MRSA

**Fig 2:**
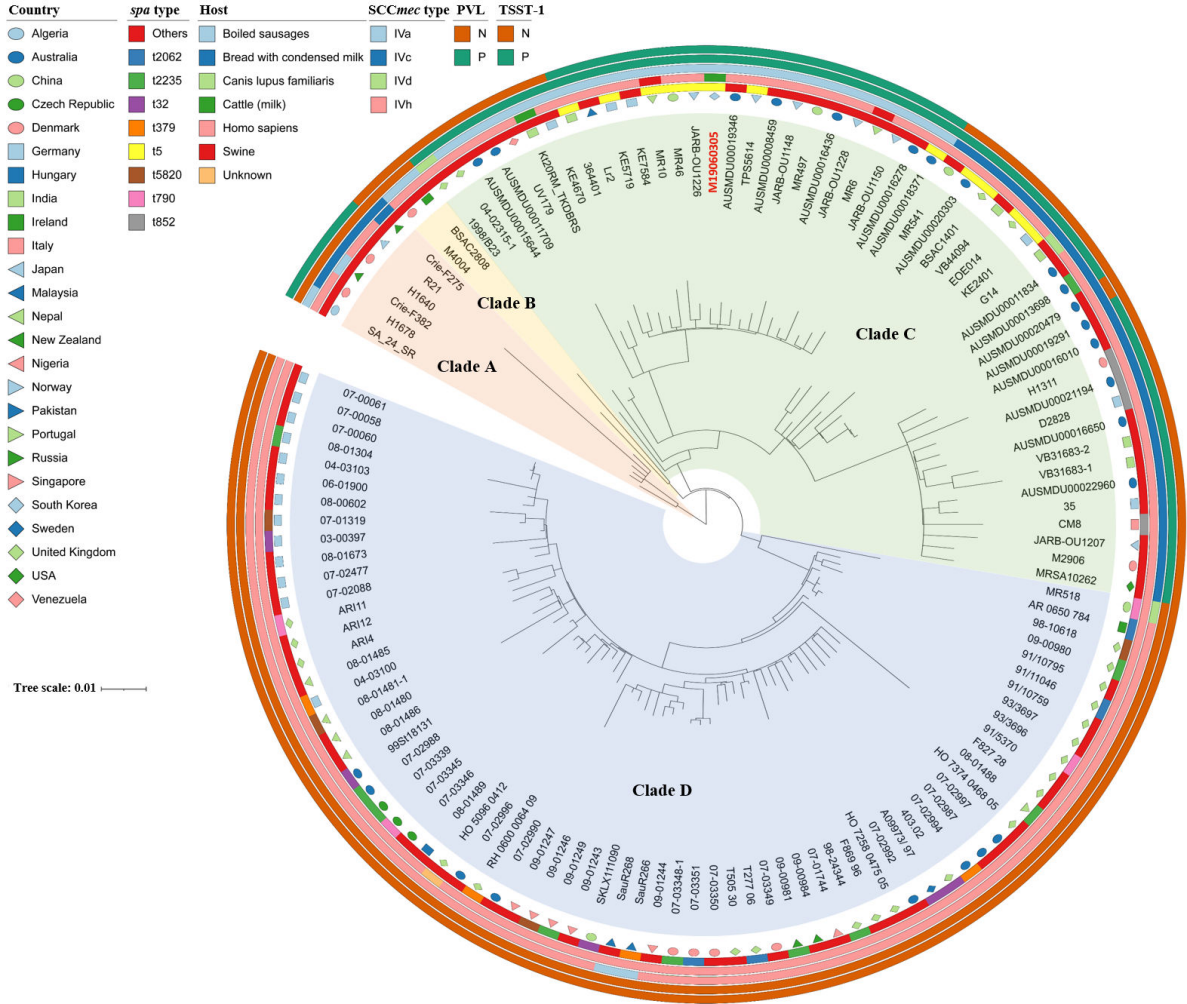
Whole-genome-based SNP phylogenetic tree of 127 MRSA ST22-SCC*mec* IV strains. The country, *spa* type, host, SCC*mec* type, and presence of *pvl* and *tsst-1* are mapped on the tree (from inner to outer circle).

Whole-genome sequencing was performed to investigate the genetic characteristics of the ST22-SCC*mec* IV MRSA strain (M19060305) isolated from South Korea. Figure 2 presents a phylogenetic tree based on single-nucleotide polymorphisms (SNPs) in the core genome of 127 MRSA ST22-SCC*mec* IV strains. MRSA strain M19060305 exhibited a close genetic relationship with MRSA AUSMDU00019346 from Australia and MRSA TPS5614 from Japan. The 127 ST22-SCC*mec* IV MRSA strains formed four major clades. Clades were divided based on various characteristics of the strains, including their geographic regions, genotypes, and virulence genes. Clade C, which included the M06190305 strain isolated in this study, has 50 strains. Notably, this clade consisted primarily of strains from Asia, specifically Japan and China. Almost all strains in Clade C harbored the PVL gene, except one. Notably, none of the SCC*mec* IVc strains in Clade C harbored *tsst-1*, whereas the majority (76.0%, 19/25) of SCC*mec* IVa strains had both *pvl* and *tsst-1*. Clade D, the largest clade, included 69 strains, the majority of which were from Europe, particularly the United Kingdom and Germany. Surprisingly, no strain in this clade harbored *pvl* or *tsst-1*, but all three (two SCC*mec* IVa and one SCC*mec* Ivd) belonged to the SCC*mec* IVh subtype. Clade A consisted of six strains, the majority of which were derived from Europe, specifically SCC*mec* IVc, with all but one strain harboring *tsst-1*. The smallest clade, Clade B, contained only two SCC*mec* IVa strains from Europe, and no strain in this clade harbored *pvl* or *tsst-1*.

## DISCUSSION

Mastitis causes substantial financial loss in the dairy industry, resulting in higher treatment costs and lower milk production (13, 14). MRSA has repeatedly been isolated from mastitis-infected dairy cows in South Korea (6, 10, 11). To develop a control or elimination policy for disease-related pathogens, understanding their general characteristics and genetic background is crucial (15). This study also examined the molecular characteristics, biofilm formation ability, virulence genes, and antimicrobial resistance of MRSA isolated from the milk of dairy cows with mastitis in South Korea. Whole-genome sequencing was performed on the PVL- and TSST-1-positive ST22-SCC*mec* IV MRSA strains.

In this study, MRSA was found in 6.1% of all *S. aureus* isolates from the quarter milk of mastitis-infected dairy cows. These findings are consistent with those of a previous study conducted in South Korea between 2003 and 2009, which reported a 6.2% distribution (10). However, this is lower than the 13.9% reported in a study conducted from 2011 to 2012 and the 15.1% reported in a study conducted from 2014 to 2018 (6, 11). Studies from other countries such as Italy, China, and Ethiopia, reported a prevalence of 11.7%, 14.2%, and 32.4% of MRSA among *S. aureus* isolates from the milk of dairy cows with mastitis, respectively, which is considerably higher than the rates reported in this study (3, 13, 16). These variations in the MRSA distribution in dairy cow mastitis may be attributed to differences in farm management practices, antimicrobial utilization patterns, and localized epidemiological impacts (3, 7, 16).

Although the global issue of antibiotic residue and resistance owing to overuse has become a major concern, antibiotics remain the primary treatment for mastitis (1). Resistance to aminoglycoside antimicrobials is mostly conferred by aminoglycoside-modifying enzymes encoded by plasmids or transposons (1). The *ant(4′)-Ia* gene, encoding aminoglycoside-4′-O-nucleotidyl transferase I, confers resistance to neomycin, kanamycin, tobramycin, and amikacin (1). As previously stated, MRSA isolates had the highest resistance rate (66.7%) to kanamycin among non-beta-lactam antibiotics (*P* < 0.05). Our results are consistent with those of the previous studies conducted in South Korea, where MRSA isolates from milk from cows with mastitis were highly resistant to kanamycin (6, 9). In this study, the high kanamycin resistance of MRSA isolates may be attributed to the aminoglycoside resistance genes *ant(4′)-Ia* (70.0%) and *aac(6′)-Ie + aph(2″)* (40.0%). Notably, all of the kanamycin-resistant MRSA isolates harbored one of these genes. Neomycin sulfate, an aminoglycoside, is the most commonly used antibiotic for cattle in South Korea (17). Therefore, the pronounced resistance to kanamycin in MRSA isolates may be due to its widespread use in cattle. MRSA, which has been isolated from dairy cows, is most likely transmitted via contact with dairy cows or products (18). These infections can cause severe disease owing to resistance against several antimicrobials, which complicates treatment (1, 18). Consequently, constant monitoring of MRSA on dairy farms is essential for preventing mastitis and preserving public health.

The capacity of *S. aureus* to form biofilms and attach to the mammary gland epithelium aids in immune defense evasion, antimicrobial resistance, and repeated or chronic infections (1). Insight into the biofilm generation ability of bovine mastitis-causing MRSA in South Korea is lacking. In our study, all evaluated MRSA isolates generated biofilms, with the majority being moderate or strong biofilm producers, except for one isolate that formed weak biofilms. These results are similar to those of a recent study conducted in India, which found that the majority of MRSA isolates from bovine mastitis formed strong-to-moderate biofilms (19). Earlier studies in Belgium and Italy found that all MRSA strains isolated from milk from cows with mastitis were able to form biofilms (3, 20). All MRSA isolates in this study harbored at least five biofilm-associated genes. Detection rates for the *clfA*, *clfB*, *ebpS*, *eno*, *fib*, *icaA*, and *icaD* genes exceeded 90% in the tested isolates. These results are consistent with those of a previous study conducted in India, which reported high prevalence rates of *icaA*, *icaD*, *eno*, and *ebp* genes in MRSA isolated from the milk of cattle with mastitis (14). Our findings revealed that most MRSA isolates in the milk of dairy cows with mastitis in South Korea are strong biofilm producers, thereby highlighting the need for management systems that may reduce and eliminate biofilm-producing MRSA isolates from dairy farms.

In terms of virulence risk, our findings also indicated that the MRSA isolates harbored five or more virulence genes. Enterotoxins cause inflammation and mammary tissue damage in bovine mastitis as well as foodborne diseases (1, 4). In this study, the most common enterotoxin gene cluster found in MRSA isolates was *seg + sei + sem + sen + seo*. This result is consistent with previous studies conducted in South Korea from 2011 to 2012 and 2014 to 2018 (6, 11). Hemolysins cause chronic infections in the mammary glands of dairy cows and are associated with pulmonary edema and dermonecrotic damage in humans (1, 4). Except for one, all MRSA isolates in our study contained either *hla* or *hlb*. In contrast to our findings, a prior study in northwest China reported that 13.6% and 9.1% of all MRSA isolates harbored the *hla* and *hlb* genes, respectively (13). Both PVL-encoding and TSST-encoding genes were found in four of the 30 MRSA isolates studied, which were collected from two farms in the same province. PVL, primarily composed of lukS and lukF proteins worsens staphylococcal infections in humans and is also associated with bovine mastitis in dairy ruminants (4, 7, 11). PVL, along with SCC*mec* IV, is a molecular marker of CA-MRSA (21). TSST-1 is a superantigen that causes apoptosis and cell death, resulting in lethal toxic shock (4). MRSA strains that produce both PVL and TSST-1 tend to cause more complicated infections (21). Although MRSA strains positive for PVL or TSST-1 have been detected globally, those carrying both genes are relatively rare (21, 22). In South Korea, MRSA strains carrying PVL-encoding genes were isolated in 2015 (11), whereas TSST-encoding genes were not found in MRSA isolates from the milk of dairy cows with mastitis (6, 11). Our study indicates that these MRSA isolates harbor various virulence genes and must therefore be closely monitored to prevent their spread.

Understanding the molecular properties of *S. aureus* allows monitoring of the evolution, epidemiology, and spread of clones of concern, such as hospital-, community-, and livestock-associated MRSA, the surveillance of which is critical for preserving public health (5, 23). The molecular characterization of MRSA strains revealed that the most common MRSA isolate was ST72-t324-SCC*mec* IV, which is consistent with the results of previous studies conducted in South Korea (10, 11). Notably, MRSA ST72-SCC*mec* IV is the most prevalent strain in the community and has rapidly spread to hospitals in South Korea (11, 24). In the current study, three isolates were identified as ST22-SCC*mec* IV. Notably, both PVL- and TSST-1-encoding genes were detected in these isolates. To our knowledge, this is the first report of PVL- and TSST-1-positive ST22-SCC*mec* IV in South Korea. ST22-SCC*mec* IV has been associated with mastitis in Pakistan (25). MRSA lineages that are frequently associated with humans, including ST22, are rarely detected in dairy cows (26, 27). ST22-MRSA is regarded as a clinically relevant clone because the epidemic strain EMRSA-15 belongs to ST22 and can outcompete and replace other previously epidemic MRSA clones (21, 27). Therefore, the presence of PVL- and TSST-1-positive ST22-MRSA in bovine milk represents a public health risk in South Korea.

### Conclusion

Our findings suggest that the majority of MRSA isolates in milk from dairy cows with mastitis are multidrug-resistant, capable of forming robust biofilms, and harbor multiple virulence genes, including those encoding enterotoxins, hemolysins, leukotoxins, and superantigens. Additionally, molecular typing performed in this study revealed the frequency of specific MRSA lineages, with the most prevalent being ST72-t324-SCC*mec* IV. Importantly, the appearance of CA-MRSA clones, such as the highly pathogenic PVL- and TSST-1-positive ST22-SCC*mec* IV, raises serious concern regarding mastitis control on farms and the threat this poses to public health in South Korea. Therefore, regular surveillance and monitoring of antimicrobial resistance are required to reduce the spread of MRSA on dairy farms and minimize the associated public health risks.

## MATERIALS AND METHODS

### Isolation and identification of *S. aureus*

Between 2017 and 2022, 488 *S*. *aureus* isolates were collected from 20 regional laboratories and centers in South Korea as part of the National Bovine Mastitis Prevention System. Using the National Mastitis Council protocol (28), *S. aureus* was isolated from the quarter milk of lactating cows suspected of having clinical or subclinical mastitis on a dairy farm. *S. aureus* isolates were submitted to the Animal and Plant Quarantine Agency for further characterization. For confirmation, these isolates were identified using matrix-assisted laser desorption ionization-time of flight mass spectrometry (bioMérieux, Marcy L’Etoile, France) following the manufacturer’s instructions. The isolates were preserved at −80°C.

### Antimicrobial resistance testing

*S. aureus* isolates were evaluated for antimicrobial susceptibility using the broth microdilution method on a Sensititre mastitis plate (CMV1AMAF; Trek Diagnostics, Cleveland, OH, USA) following the guidelines of the Clinical and Laboratory Standards Institute (CLSI) (29). The Sensititre mastitis plate was used to test 10 antimicrobials at varying concentrations (μg/mL): ampicillin (0.12–8), cephalothin (2–16), ceftiofur (1–4), penicillin (0.12–8), penicillin-novobiocin combination (1/2–8/16), pirlimycin (0.5–4), sulfadimethoxine (32–256), erythromycin (0.25–4), oxacillin + 2% NaCl (2–4), and tetracycline (1–8). MRSA isolates were subsequently evaluated using a Sensititre EUST plate (Trek Diagnostics). The Sensititre EUST plate was used to test 19 antimicrobials at varying concentrations (μg/mL): erythromycin (0.25–8), gentamicin (1–16), kanamycin (4–64), streptomycin (4–32), sulfamethoxazole (64–512), rifampin (0.016–0.5), ciprofloxacin (0.25–8), vancomycin (1–16), linezolid (1–8), trimethoprim (2–32), cefoxitin (0.5–16), clindamycin (0.12–4), chloramphenicol (4–64), mupirocin (0.5–256), quinupristin/dalfopristin (0.5–4), fusidate (0.5–4), penicillin (0.12–2), tetracycline (0.5–16), and tiamulin (0.5–4). Susceptibility data were evaluated using the criteria described in the CLSI M100, CLSI VET08, European Committee on Antimicrobial Susceptibility Testing guidelines, and Saini et al. (29–32). *S. aureus* ATCC 25913 served as a quality control strain.

### Molecular typing of MRSA

MLST was performed using the method described by Enright et al. (33). The primer sequences for seven housekeeping genes (*arcC*, *aroE*, *glpF*, *gmk*, *pta*, *tpi*, and *yqiL*) were constructed following the instructions on the *S. aureus* multilocus sequence typing website (https://pubmlst.org/organisms/staphylococcus-aureus/primers). Further, *spa* type was identified using previously established protocols (34) and the coding system described on the Ridom SpaServer website (www.spa.ridom.de). MRSA isolates were characterized for SCC*mec* using multiplex PCR as previously described (35). PFGE of MRSA genomic DNA digested with *SmaI* was performed as previously described (36). The resulting patterns were analyzed using Bionumerics v8.0 (Applied Maths, Sint-Martens-Latem, Belgium), producing a dendrogram via the unweighted pair group method with arithmetic mean and a dice similarity coefficient of 1.5% position tolerance.

### Detection of biofilm-, virulence-, and antimicrobial resistance-related genes

MRSA isolates were examined for the presence of genes involved in biofilm formation, virulence, and antimicrobial resistance. DNA was extracted using the boiling method, as previously described (37). PCR was used to detect various genes in MRSA, as previously described. The genes tested included 12 biofilm-related genes (*bap*, *bbp*, *clfA*, *clfB*, *cna*, *ebpS*, *eno*, *fib*, *fnbA*, *fnbB*, *icaA*, and *icaD*) (38–41), 24 virulence genes (*eta*, *etb*, *hla*, *hlb*, *lukED*, *pvl*, *sea*, *seb*, *sec*, *sed*, *see*, *seg*, *seh*, *sei*, *sek*, *sel*, *sem*, *sen*, *seo*, *sep*, *seq*, *ser*, and *tsst1*) (42–47), and 13 antibiotic resistance genes (*aac(6′)-Ie*, *aph(2*″), *ant(4′)-Ia*, *aph(3′)-IIIa*, *blaZ*, *cfr*, *ermA*, *ermB*, *ermC*, *fexA*, *mecA*, *msrA*, *tetK*, and *tetM*) (48–51).

### Biofilm formation testing

The potential of MRSA isolates to form biofilms was determined using a 96-well microtiter plate assay, as previously described by Kim et al. (36). Isolates exhibiting the capacity to produce biofilms were classified as follows: non-adherent [OD ≤ OD negative control (NC)], weak (OD NC < OD ≤ 2 × OD NC), moderate (2 × OD NC < OD ≤ 4 × OD NC), or strong (OD > 4 × OD NC).

### Whole-genome sequencing and comparative genomics analysis

The genomic DNA of the MRSA M19060305 strain was sequenced using the Oxford Nanopore GridION (Oxford Nanopore Technologies, Oxford, UK) and Illumina NextSeq 2000 platforms (Illumina, San Diego, CA, USA). Nanopore sequencing was performed using a ligation sequencing kit, and base calling was performed using Guppy v4.4.2 (Oxford Nanopore Technologies), followed by filtering using Nanofilt v2.8.0 (Purdue University, West Lafayette, IN, USA) to remove reads with a *Q*-score of <7 and a minimum length of <1,000 bases. Illumina sequencing was performed using the TruSeq Nano DNA Prep Kit (Illumina), and reads were filtered using Trimmomatic v0.39 with a *Q*-score threshold of 20. Hybrid assembly was performed using Unicycler v0.5.0, and the final genome was annotated using Prokka v1.14.6 (52).

To determine the evolutionary relationships among the ST22-SCC*mec* IV strains, a comparative analysis of the publicly accessible genomes of 126 ST22-SCC*mec* IV MRSA strains and the genome of MRSA strain M19060305, isolated in our study, was performed (Table S1). One hundred twenty-six genome sequences of ST22-SCC*mec* IV MRSA strains were obtained from GenBank. The SCCmecFinder, spaTyper, and VirulenceFinder databases, available on the Center for Genomic Epidemiology website (https://genomicepidemiology.org/), were used to type the 127 MRSA strains as well as screen for virulence genes. The PanX pipeline with the default parameters was used to analyze core genome SNPs and generated a maximum likelihood phylogenetic tree (53). The resulting phylogenetic tree was visualized, and iTOL (https://itol.embl.de/) was used to create data on the isolated nation, host, *spa* type, SCC*mec* type, and presence or absence of virulence genes in the strains.

### Statistical analysis

Statistical analyses were performed using the Statistical Package for the Social Sciences version 25 (IBM SPSS Statistics for Windows, Armonk, NY, USA). The analyses included independent samples *t* tests, Fisher’s exact tests, and Pearson’s chi-square tests, with a Bonferroni correction applied. A significance level of *P* < 0.05 was used for determining statistically significant differences.

## Data Availability

The data sets presented in this paper can be found in online repositories. The names of the repositories and accession numbers are available at https://www.ncbi.nlm.nih.gov/, PRJNA1063000.

## SUPPLEMENTAL MATERIAL

Table S1 spectrum.01197-24-s0001.docx Information about the 127 MRSA ST22-SCCmec IV strains used in the comparative analysis.

ASM does not own the copyrights to Supplemental Material that may be linked to, or accessed through, an article. The authors have granted ASM a non-exclusive, world-wide license to publish the Supplemental Material files. Please contact the corresponding author directly for reuse.
