# Supplementary material for: Characteristics of methicillin-resistant Staphylococcus aureus isolates from bovine mastitis milk in South Korea: molecular characteristics, biofilm, virulence, and antimicrobial resistance
Source: Microbiol Spectr. 2024 Oct 22;12(12):e01197-24. doi: 10.1128/spectrum.01197-24 (PMC11619599; doi:10.1128/spectrum.01197-24)
Supplement: Table S1 — Information about the 127 MRSA ST22-SCCmec IV strains used in the comparative analysis. [file spectrum.01197-24-s0001.docx]

**Table S1** Information about the 127 MRSA ST22-SCC*mec* IV strains used in the comparative analysis

| No. | GenBank accession | Strain | Host | Year of isolation | Country | *spa* type | SCC*mec* type | PVL | TSST-1 | Clade |
| --- | --- | --- | --- | --- | --- | --- | --- | --- | --- | --- |
| 1 | GCA_023621705.1 | SA_24_SR | Homo sapiens | 2021 | Algeria | t3243 | IVa | N | P | A |
| 2 | GCA_900250645.1 | H1678 | Homo sapiens | 2014 | Denmark | t223 | IVc | N | P | A |
| 3 | GCA_022899495.1 | Crie-F382 | Boiled sausages | 2019 | Russia | t223 | IVc | N | P | A |
| 4 | GCA_900250565.1 | H1640 | Homo sapiens | 2014 | Denmark | t12437 | IVc | N | P | A |
| 5 | GCA_014337055.1 | R21 | Homo sapiens | 2012 | Norway | t223 | IVc | N | P | A |
| 6 | GCA_022899315.1 | Crie-F275 | Bread with condensed milk | 2019 | Russia | t2571 | IVc | N | N | A |
| 7 | GCA_900251985.1 | M4004 | Homo sapiens | 2014 | Denmark | t1328 | IVa | N | N | B |
| 8 | GCA_900051775.1 | BSAC2808 | Homo sapiens | 2010 | Ireland | t1132 | IVa | N | N | B |
| 9 | GCA_001224085.1 | 1998/B23 | Homo sapiens | 1998 | United Kingdom | t474 | IVd | P | N | C |
| 10 | GCA_001227645.1 | 04-02315-1 | Homo sapiens | 2004 | United Kingdom | t310 | IVa | P | N | C |
| 11 | GCA_017663035.1 | AUSMDU00015644 | Homo sapiens | 2018 | Australia | t2518 | IVa | P | N | C |
| 12 | GCA_017661495.1 | AUSMDU00011709 | Homo sapiens | 2017 | Australia | t891 | IVa | P | N | C |
| 13 | GCA_002887415.1 | UV179 | Homo sapiens | 2012 | Venezuela | NA | IVa | P | N | C |
| 14 | GCA_016888235.1 | Kt20RM_TKDBRS | Cattle (milk) | 2017 | India | t1328 | IVa | P | N | C |
| 15 | GCA_014181175.1 | KE4670 | Homo sapiens | 2017 | Germany | t1328 | IVa | P | N | C |
| 16 | GCA_027717345.1 | 364401 | Homo sapiens | 2021 | India | t5 | IVa | P | P | C |
| 17 | GCA_005706855.1 | Lr2 | Homo sapiens | 2017 | Pakistan | t2986 | IVa | P | P | C |
| 18 | GCA_014181085.1 | KE5719 | Homo sapiens | 2017 | Germany | t5 | IVa | P | P | C |
| 19 | GCA_014181055.1 | KE7584 | Homo sapiens | 2018 | Germany | t417 | IVa | P | P | C |
| 20 | GCA_002843805.1 | MR10 | Swine | 2016 | Nepal | t5 | IVa | P | P | C |
| 21 | GCA_029853615.1 | MR46 | Homo sapiens | 2019 | China | t5 | IVa | P | P | C |
| 22 | GCA_023706925.1 | JARB-OU1226 | Homo sapiens | 2018 | Japan | t5 | IVa | P | P | C |
| **23** | **This study** | **M19060305** | **Cattle (milk)** | **2019** | **South Korea** | **t5** | **IVa** | **P** | **P** | **C** |
| 24 | GCA_017092345.1 | AUSMDU00019346 | Homo sapiens | 2018 | Australia | t18929 | IVa | P | P | C |
| 25 | GCA_020887155.1 | TPS5614 | Homo sapiens | 2019 | Japan | t5 | IVa | P | P | C |
| 26 | GCA_015600465.1 | AUSMDU00008459 | Homo sapiens | 2017 | Australia | t3638 | IVa | P | P | C |
| 27 | GCA_023706465.1 | JARB-OU1148 | Homo sapiens | 2018 | Japan | t13828 | IVa | P | P | C |
| 28 | GCA_029853355.1 | MR497 | Homo sapiens | 2020 | China | t309 | IVa | P | P | C |
| 29 | GCA_017098445.1 | AUSMDU00016436 | Homo sapiens | 2018 | Australia | t309 | IVa | P | P | C |
| 30 | GCA_023706965.1 | JARB-OU1228 | Homo sapiens | 2018 | Japan | NA | IVa | P | P | C |
| 31 | GCA_002843855.1 | MR6 | Swine | 2016 | Nepal | t309 | IVa | P | P | C |
| 32 | GCA_023706505.1 | JARB-OU1150 | Homo sapiens | 2018 | Japan | t845 | IVa | P | P | C |
| 33 | GCA_017098785.1 | AUSMDU00016278 | Homo sapiens | 2018 | Australia | t458 | IVa | P | P | C |
| 34 | GCA_017099895.1 | AUSMDU00018371 | Homo sapiens | 2018 | Australia | t5 | IVa | P | P | C |
| 35 | GCA_029853095.1 | MR541 | Homo sapiens | 2020 | China | t474 | IVc | P | N | C |
| 36 | GCA_017088585.1 | AUSMDU00020303 | Homo sapiens | 2018 | Australia | t5 | IVc | P | N | C |
| 37 | GCA_900018285.1 | BSAC1401 | Homo sapiens | 2006 | United Kingdom | t5 | IVc | P | N | C |
| 38 | GCA_001855035.1 | VB44094 | Homo sapiens | 2015 | India | t474 | IVc | P | N | C |
| 39 | GCA_900482565.1 | EOE014 | Homo sapiens | 2010 | United Kingdom | t5 | IVc | P | N | C |
| 40 | GCA_014181425.1 | KE2401 | Homo sapiens | 2016 | Germany | t5 | IVc | P | N | C |
| 41 | GCA_015209565.1 | G14 | Canis lupus familiaris | 2018 | India | t2518 | IVc | P | N | C |
| 42 | GCA_017664375.1 | AUSMDU00011834 | Homo sapiens | 2018 | Australia | t3002 | IVc | P | N | C |
| 43 | GCA_017668435.1 | AUSMDU00013698 | Homo sapiens | 2018 | Australia | t2235 | IVc | P | N | C |
| 44 | GCA_017088845.1 | AUSMDU00020479 | Homo sapiens | 2018 | Australia | t3002 | IVc | N | N | C |
| 45 | GCA_017093285.1 | AUSMDU00019291 | Homo sapiens | 2018 | Australia | t3107 | IVc | P | N | C |
| 46 | GCA_017664215.1 | AUSMDU00016010 | Homo sapiens | 2017 | Australia | t605 | IVc | P | N | C |
| 47 | GCA_900250295.1 | H1311 | Homo sapiens | 2014 | Denmark | t852 | IVc | P | N | C |
| 48 | GCA_017083605.1 | AUSMDU00021194 | Homo sapiens | 2018 | Australia | t852 | IVc | P | N | C |
| 49 | GCA_014181715.1 | D2828 | Homo sapiens | 2016 | Germany | t852 | IVc | P | N | C |
| 50 | GCA_017101985.1 | AUSMDU00016650 | Homo sapiens | 2018 | Australia | NA | IVc | P | N | C |
| 51 | GCA_004153365.1 | VB31683-2 | Homo sapiens | 2015 | India | t1317 | IVc | P | N | C |
| 52 | GCA_001756665.1 | VB31683-1 | Homo sapiens | 2015 | India | NA | IVc | P | N | C |
| 53 | GCA_017091345.1 | AUSMDU00022960 | Homo sapiens | 2018 | Australia | t4573 | IVc | P | N | C |
| 54 | GCA_015604125.1 | 35 | Homo sapiens | 2015 | Germany | t4573 | IVc | P | N | C |
| 55 | GCA_003238995.1 | CM8 | Homo sapiens | 2013 | Italy | t852 | IVc | P | N | C |
| 56 | GCA_023706585.1 | JARB-OU1207 | Homo sapiens | 2018 | Japan | t4573 | IVc | P | N | C |
| 57 | GCA_900250995.1 | M2906 | Homo sapiens | 2014 | Denmark | t4326 | IVc | P | N | C |
| 58 | GCA_015729265.1 | MRSA10262 | Homo sapiens | 2017 | USA | NA | IVc | P | N | C |
| 59 | GCA_029853085.1 | MR518 | Homo sapiens | 2019 | China | t790 | IVd | N | N | D |
| 60 | GCA_001229205.1 | AR 0650 784 | Homo sapiens | 1993 | Ireland | t2062 | IVh | N | N | D |
| 61 | GCA_001234265.1 | 98-10618 | Homo sapiens | 1998 | United Kingdom | t5820 | IVh | N | N | D |
| 62 | GCA_001229925.1 | 09-00980 | Homo sapiens | 1996 | United Kingdom | t2235 | IVh | N | N | D |
| 63 | GCA_001223785.1 | 91/10795 | Homo sapiens | 1991 | United Kingdom | t14857 | IVh | N | N | D |
| 64 | GCA_001238225.1 | 91/11046 | Homo sapiens | 1991 | United Kingdom | t2062 | IVh | N | N | D |
| 65 | GCA_001226725.1 | 91/10759 | Homo sapiens | 1991 | United Kingdom | t22 | IVh | N | N | D |
| 66 | GCA_001237045.1 | 93/3697 | Homo sapiens | 1993 | United Kingdom | t2892 | IVh | N | N | D |
| 67 | GCA_001225765.1 | 93/3696 | Homo sapiens | 1993 | United Kingdom | t790 | IVh | N | N | D |
| 68 | GCA_001228345.1 | 91/5370 | Homo sapiens | 1991 | United Kingdom | NA | IVh | N | N | D |
| 69 | GCA_001224445.1 | F827 28 | Homo sapiens | 2007 | United Kingdom | t1258 | IVh | N | N | D |
| 70 | GCA_001232805.1 | 08-01488 | Homo sapiens | 2006 | Portugal | t2235 | IVh | N | N | D |
| 71 | GCA_001232845.1 | HO 7374 0468 05 | Homo sapiens | 2007 | United Kingdom | t1437 | IVh | N | N | D |
| 72 | GCA_001232185.1 | 07-02997 | Homo sapiens | 2006 | Australia | t3212 | IVh | N | N | D |
| 73 | GCA_001233885.1 | 07-02987 | Homo sapiens | 2006 | Australia | t3010 | IVh | N | N | D |
| 74 | GCA_001236985.1 | 07-02994 | Homo sapiens | 2006 | Australia | t379 | IVh | N | N | D |
| 75 | GCA_001238005.1 | 403.02 | Homo sapiens | 2002 | United Kingdom | t32 | IVh | N | N | D |
| 76 | GCA_001236005.1 | A09973/ 97 | Homo sapiens | 1997 | Sweden | t32 | IVh | N | N | D |
| 77 | GCA_001224045.1 | 07-02992 | Homo sapiens | 2006 | Australia | NA | IVh | N | N | D |
| 78 | GCA_001231865.1 | HO 7258 0475 05 | Homo sapiens | 2007 | United Kingdom | t910 | IVh | N | N | D |
| 79 | GCA_001226685.1 | F869 96 | Homo sapiens | 2007 | United Kingdom | t1461 | IVh | N | N | D |
| 80 | GCA_001235725.1 | 98-24344 | Homo sapiens | 1998 | United Kingdom | t2235 | IVh | N | N | D |
| 81 | GCA_001223645.1 | 07-01744 | Homo sapiens | 2002 | Nigeria | NA | IVh | N | N | D |
| 82 | GCA_001228885.1 | 09-00984 | Homo sapiens | 1996 | New Zealand | NA | IVh | N | N | D |
| 83 | GCA_001226205.1 | 09-00981 | Homo sapiens | 1996 | New Zealand | t2235 | IVh | N | N | D |
| 84 | GCA_001236485.1 | 07-03349 | Homo sapiens | 2005 | Denmark | NA | IVh | N | N | D |
| 85 | GCA_001230185.1 | T277 06 | Homo sapiens | 2008 | United Kingdom | t2062 | IVh | N | N | D |
| 86 | GCA_001229865.1 | T505 30 | Homo sapiens | 2007 | United Kingdom | t22 | IVh | N | N | D |
| 87 | GCA_001225965.1 | 07-03350 | Homo sapiens | 2005 | Denmark | t1895 | IVh | N | N | D |
| 88 | GCA_001229525.1 | 07-03351 | Homo sapiens | 2005 | Denmark | t2062 | IVh | N | N | D |
| 89 | GCA_001224685.1 | 07-03348-1 | Homo sapiens | 2004 | Denmark | t2235 | IVh | N | N | D |
| 90 | GCA_001232345.1 | 09-01244 | Homo sapiens | 2007 | Singapore | t1802 | IVh | N | N | D |
| 91 | GCA_020297225.1 | SauR266 | Homo sapiens | 2020 | Malaysia | t379 | IVa | N | N | D |
| 92 | GCA_020166275.1 | SauR268 | Homo sapiens | 2020 | Malaysia | NA | IVa | N | N | D |
| 93 | GCA_021404045.1 | SKLX111090 | Homo sapiens | 2019 | China | t32 | IVh | N | N | D |
| 94 | GCA_001237225.1 | 09-01243 | Homo sapiens | 2004 | Singapore | NA | IVh | N | N | D |
| 95 | GCA_001234845.1 | 09-01249 | Homo sapiens | 2007 | Singapore | t2235 | IVh | N | N | D |
| 96 | GCA_001234985.1 | 09-01246 | Homo sapiens | 2007 | Singapore | t5820 | IVh | N | N | D |
| 97 | GCA_001229845.1 | 09-01247 | Homo sapiens | 2007 | Singapore | NA | IVh | N | N | D |
| 98 | GCA_001234705.1 | 07-02990 | Homo sapiens | 2006 | Australia | t458 | IVh | N | N | D |
| 99 | GCA_001224765.1 | RH 0600 0064 09 | Homo sapiens | 2005 | United Kingdom | t379 | IVh | N | N | D |
| 100 | GCA_001238025.1 | 07-02996 | Homo sapiens | 2006 | Australia | t1977 | IVh | N | N | D |
| 101 | GCA_000284535.1 | HO 5096 0412 | Unknown | 2005 | United Kingdom | t1041 | IVh | N | N | D |
| 102 | GCA_001236585.1 | 08-01489 | Homo sapiens | 2003 | Hungary | NA | IVh | N | N | D |
| 103 | GCA_001227105.1 | 07-03346 | Homo sapiens | 2005 | Czech Republic | NA | IVh | N | N | D |
| 104 | GCA_001234645.1 | 07-03345 | Homo sapiens | 2005 | Czech Republic | t790 | IVh | N | N | D |
| 105 | GCA_001227025.1 | 07-03339 | Homo sapiens | 2006 | Czech Republic | t2235 | IVh | N | N | D |
| 106 | GCA_001235225.1 | 07-02988 | Homo sapiens | 2006 | Australia | t2235 | IVh | N | N | D |
| 107 | GCA_001233485.1 | 99St18131 | Homo sapiens | 1999 | Australia | t32 | IVh | N | N | D |
| 108 | GCA_001230065.1 | 08-01486 | Homo sapiens | 2006 | Portugal | t819 | IVh | N | N | D |
| 109 | GCA_001226165.1 | 08-01480 | Homo sapiens | 2006 | Portugal | t1895 | IVh | N | N | D |
| 110 | GCA_001224925.1 | 08-01481-1 | Homo sapiens | 2006 | Portugal | t5820 | IVh | N | N | D |
| 111 | GCA_001238145.1 | 04-03100 | Homo sapiens | 2003 | Germany | t379 | IVh | N | N | D |
| 112 | GCA_001226305.1 | 08-01485 | Homo sapiens | 2006 | Portugal | NA | IVh | N | N | D |
| 113 | GCA_001231805.1 | ARI4 | Homo sapiens | 2007 | United Kingdom | t1214 | IVh | N | N | D |
| 114 | GCA_001228065.1 | ARI12 | Homo sapiens | 2007 | United Kingdom | t2892 | IVh | N | N | D |
| 115 | GCA_001226565.1 | ARI11 | Homo sapiens | 2007 | United Kingdom | t790 | IVh | N | N | D |
| 116 | GCA_001225945.1 | 07-02088 | Homo sapiens | 2007 | Germany | NA | IVh | N | N | D |
| 117 | GCA_001228925.1 | 07-02477 | Homo sapiens | 2007 | Germany | t3212 | IVh | N | N | D |
| 118 | GCA_001224605.1 | 08-01673 | Homo sapiens | 2007 | Germany | t531 | IVh | N | N | D |
| 119 | GCA_001229165.1 | 03-00397 | Homo sapiens | 2003 | Germany | t32 | IVh | N | N | D |
| 120 | GCA_001228385.1 | 07-01319 | Homo sapiens | 2007 | Germany | t5820 | IVh | N | N | D |
| 121 | GCA_001226445.1 | 08-00602 | Homo sapiens | 2008 | Germany | t1802 | IVh | N | N | D |
| 122 | GCA_001226365.1 | 06-01900 | Homo sapiens | 2006 | Germany | t845 | IVh | N | N | D |
| 123 | GCA_001229005.1 | 04-03103 | Homo sapiens | 2004 | Germany | t1214 | IVh | N | N | D |
| 124 | GCA_001237765.1 | 08-01304 | Homo sapiens | 2008 | Germany | t2235 | IVh | N | N | D |
| 125 | GCA_001239525.1 | 07-00060 | Homo sapiens | 2006 | Germany | t8195 | IVh | N | N | D |
| 126 | GCA_001232445.1 | 07-00058 | Homo sapiens | 2006 | Germany | t1977 | IVh | N | N | D |
| 127 | GCA_001228765.1 | 07-00061 | Homo sapiens | 2006 | Germany | t1437 | IVh | N | N | D |

NA, Not available; N, Negative; P, Positive.
